# Inhibition of Sesn2 has negative regulatory effects on the myogenic differentiation of C2C12 myoblasts

**DOI:** 10.1186/s43556-024-00193-z

**Published:** 2024-08-09

**Authors:** Zubiao Song, Qing Lin, Jiahui Liang, Weixi Zhang

**Affiliations:** Department of Neurology, The First Affiliated Hospital, Sun Yat-Sen University; Guangdong Provincial Key Laboratory of Diagnosis and Treatment of Major Neurological Diseases; National Key Clinical Department and Key Discipline of Neurology, No. 58 Zhongshan Road 2, Guangzhou, 510080 China

**Keywords:** Sestrin2, miR-182-5p, Myogenic differentiation, Duchenne muscular dystrophy, Slow-twitch myofibers

## Abstract

Sestrin2 (Sesn2) has been previously confirmed to be a stress-response molecule. However, the influence of Sesn2 on myogenic differentiation remains elusive. This study was conducted to analyze the role of Sesn2 in the myogenic differentiation of C2C12 myoblasts and related aspects in mdx mice, an animal model of Duchenne muscular dystrophy (DMD). Our results showed that knockdown of Sesn2 reduced the myogenic differentiation capacity of C2C12 myoblasts. Predictive analysis from two databases suggested that miR-182-5p is a potential regulator of Sesn2. Further experimental validation revealed that overexpression of miR-182-5p decreased both the protein and mRNA levels of Sesn2 and inhibited myogenesis of C2C12 myoblasts. These findings suggest that miR-182-5p negatively regulates myogenesis by repressing Sesn2 expression. Extending to an in vivo model of DMD, knockdown of Sesn2 led to decreased Myogenin (Myog) expression and increased Pax7 expression, while its overexpression upregulated Myog levels and enhanced the proportion of slow-switch myofibers. These findings indicate the crucial role of Sesn2 in promoting myogenic differentiation and skeletal muscle regeneration, providing potential therapeutic targets for muscular dystrophy.

**Supplementary Information:**

The online version contains supplementary material available at 10.1186/s43556-024-00193-z.

## Introduction

The sestrin (Sesn) family (Sesn1, 2, 3) is identified as a conventional group of stress-responsive proteins that modulate metabolism by monitoring nutrient levels and redox status in cells, tissues and organs [1]. Within this protein family, Sesn2 has been shown to be associated with musculoskeletal disorders [2]. In denervated muscle atrophy, Sesn2 acts to prevent the switch from slow-twitch to fast-twitch myofibers and maintains muscle mass through the AMPK/PGC- 1α pathway [3]. A study in a dexamethasone-induced muscle atrophy model also demonstrated that Sesn2 functions as a potential protective molecule by inhibiting the FoxO3a and MSTN/Smad pathways [4]. Thus, these compelling evidence have shown that Sesn2 is essential for preventing skeletal muscle atrophy. Skeletal muscle atrophy is closely related to its regenerative capacity, but the role of Sesn2 in the regeneration of skeletal muscle remains largely unknown.

Skeletal muscle inherently possesses a regenerative mechanism that maintains structural and functional stability when it is damaged by external factors or disease. Notably, satellites cells (SCs), located in the niche between the surface of the sarcolemma and the basal lamina [5], play a key role in this repair process by undergoing asymmetric division and myogenic differentiation [6]. Under quiescent conditions, SCs typically express the characteristic marker paired box 7 (Pax7), which is essential for maintaining the SCs pool and regulating their survival, proliferation, and differentiation [7, 8]. When skeletal muscle is damaged, SCs re-enter the cell cycle, proliferate and undergo myogenic differentiation to repair the damaged muscle region [9]. The impaired myogenic differentiation ability of SCs diminishes the regenerative capacity of skeletal muscles after injury, eventually causing skeletal muscle atrophy and related issues. Therefore, understanding the mechanism that influences the myogenesis of SCs would aid in promoting skeletal muscle regeneration. To conveniently uncover the factors regulating the myogenic differentiation, C2C12 myoblasts are commonly used as an in vitro model to recapitulate the differentiation process of SCs [10]. Despite numerous studies indicating that Sesn2 regulates cellular processes such as lipid biosynthesis [11], inflammation [12], and insulin resistance [13] in C2C12 cells so far, its role in myogenic differentiation remains elusive and warrant further investigation.

After leaving the cell cycle, the C2C12 myoblasts enter the myogenic differentiation program, which is mainly governed by two categories of transcription factors [14, 15]: myogenic regulatory factors (MRFs, including MyoD, Myogenin (Myog), Myf5 and MRF4 [16, 17]) and myocyte enhancer factor 2 (MEF2). MRFs, as a member of the basic helix-loop-helix (bHLH) transcription factor family, combine with E proteins such as E12 to promote the transcription of skeletal muscle-specific genes by binding to the E-box (CANNTG) within their regulatory regions [18]. Myf5 and MyoD are essential for myogenic determination [19], whereas Myog shares a structural similarity with MyoD and plays a crucial role in terminal differentiation [17, 20]. Previously, researchers inferred that Sesn2 could regulate the myogenesis of C2C12 myoblasts by controlling mitochondrial quality under stress conditions induced by reactive oxygen species (ROS), but no direct studies have confirmed this involvement [21]. Additionally, it remains unclear whether Sesn2 is regulated by other factors during the differentiation of C2C12 myoblasts.

Recent studies [22, 23] have shown that microRNAs (miRNAs) can regulate the myogenic differentiation of C2C12 cells by modulating the activity of target genes. MiRNAs, non-coding RNAs of approximately 22nt, bind to complementary sequences within the 3’UTR of target genes, leading to either degeneration of the target mRNA or the suppression of its translation [24]. For instance, miR-696 inhibits C2C12 myoblasts myogenesis by repressing CNTFR α expression [22], while miR-204-5p inhibits myogenic differentiation of C2C12 myoblasts and the formation of slow-twitch myofibers by targeting MEF2C and ERR $\gamma$ [23]. Therefore, understanding the regulatory mechanism of miRNAs not only elucidates the molecular basis underlying myoblasts differentiation but also provides new strategies for the treatment of muscular diseases.

Duchenne muscular dystrophy (DMD) is a lethal neuromuscular disorder caused by mutations in the *DMD* gene located on the X chromosome [25]. These mutations result in the loss of dystrophin, which is a component of the dystrophin-associated protein complex (DAPC) [25]. Since dystrophin is crucial for maintaining the integrity of the sarcolemma, its absence results in pathological manifestations, including myofibrillar degeneration, inflammatory infiltration and fibrosis in skeletal muscle [25].

In this study, we first investigated the effects of Sesn2 on the myogenic differentiation of C2C12 myoblasts. Additionally, we employed databases to predict miRNAs that could interact with Sesn2 mRNA and validated this regulatory relationship. Furthermore, we examined the impact of Sesn2 on the regeneration degree and the proportion of slow-twitch myofibers in skeletal muscle in DMD model mice (mdx mice). This study clarified the basis by which Sesn2 and related miRNA regulate myogenic differentiation, providing new targets for the treatment of muscular disorders such as DMD.

## Results

### Sesn2 is involved in the myogenic differentiation of C2C12 myoblasts

**Fig. 1:**
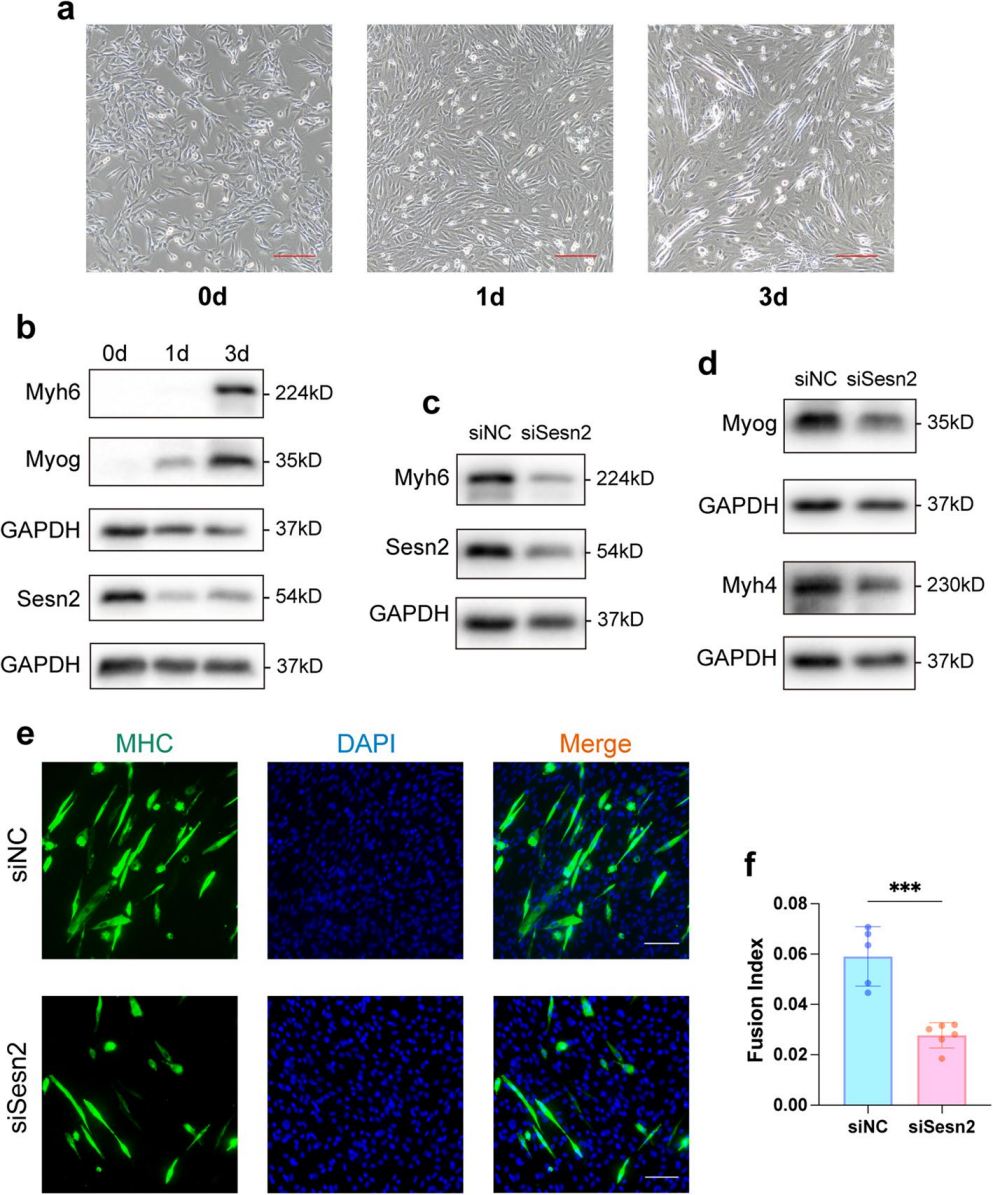
Effect of Sesn2 on the myogenic differentiation of C2C12 myoblasts. **a** Changes in cellular morphology. With increasing differentiation time, the cells gradually adopted a spindle-shaped myotube morphology. Scale bar = 200μm. **b** Temporal expression trends of myogenic differentiation markers and Sesn2. Myh6 and Myog expression gradually increased with prolongation of differentiation. However, the Sesn2 protein level significantly decreased 1 day after induction and then increased on the third day. The expression levels on days 1 and 3 were significantly lower than those in undifferentiated cells. **c**, **d** Determination of myogenic differentiation ability after Sesn2 knockdown. WB analysis revealed significant decreases in the expression of Myh6, Sesn2, Myh4 and Myog in the siSesn2 group. **e f** IF analysis of the fusion index of MHC-positive myotubes. The fusion index was significantly lower in the siSesn2 group than in the siNC group. Scale bar = 100μm. Student’s t test. The data are displayed as the mean $\pm$ standard deviation. *n* = 5–6. ***p < 0.001

To validate the effects of Sesn2 on myogenic differentiation, we first aimed to observe the expression trend of Sesn2 over the extended differentiation period of C2C12 myoblasts. On day 0 (the undifferentiated state), C2C12 myoblasts exhibited a spindle-shaped morphology (Fig. 1a). As differentiation progressed, the cells underwent a gradual morphological transformation, manifesting as the formation of distinctive elongated myotube structures indicative of successful differentiation (Fig. 1a). The expression of the Myog and Myh6 gradually increased with prolonged differentiation (Figs. 1b, S1a and S1b), further confirming differentiation toward myotube formation. Sesn2 protein expression was significantly decreased 1 day after differentiation induction (Figs. 1b and S1c). However, after 3 days of differentiation, Sesn2 expression was markedly increased compared to that on the first day, although it remained significantly lower than that in the undifferentiated cells (Day 0) (Figs. 1b and S1c).

Next, C2C12 myoblasts were divided into two groups: the siSesn2 group (transfected with Sesn2-specific siRNA) and the siNC group (transfected with siNC). After 12 h of transfection, the cells were induced to differentiate for 3 days. Western blotting (WB) analysis confirmed that the relative expression level of Sesn2 was significantly lower in the siSesn2 group than in the siNC group (0.58 $\pm$ 0.10 vs. 0.96 $\pm$ 0.15, *p* < 0.001; Figs. 1c and S1e). Subsequent comparison revealed that the relative expression of Myog in the siSesn2 group was significantly decreased by 27.93% compared with that in the siNC group (0.80 $\pm$ 0.13 vs. 1.11 $\pm$ 0.29, *p* < 0.05; Figs. 1d and S1f). Additionally, the relative expression of Myh6 in the siSesn2 group was 41.54% lower than that in the siNC group (0.38 $\pm$ 0.10 vs. 0.65 $\pm$ 0.13, *p* < 0.01; Figs. 1c and S1d), and the relative expression of Myh4 in the siSesn2 group was also lower than that in the siNC group (0.27 $\pm$ 0.18 vs. 0.66 $\pm$ 0.21, *p* < 0.01; Figs. 1d and S1g). Further immunofluorescence (IF) analysis of MHC expression revealed a significantly lower fusion index in MHC-positive myotubes in the siSesn2 group than in the siNC group (*p* < 0.001; Fig. 1e and f). These findings further confirm the involvement of Sesn2 in the myogenic differentiation of C2C12 myoblasts, and suggest that inhibiting Sesn2 expression reduced the myogenic capability.

### Predictive identification of miR-182-5p as a potential regulator of Sesn2

**Fig. 2:**
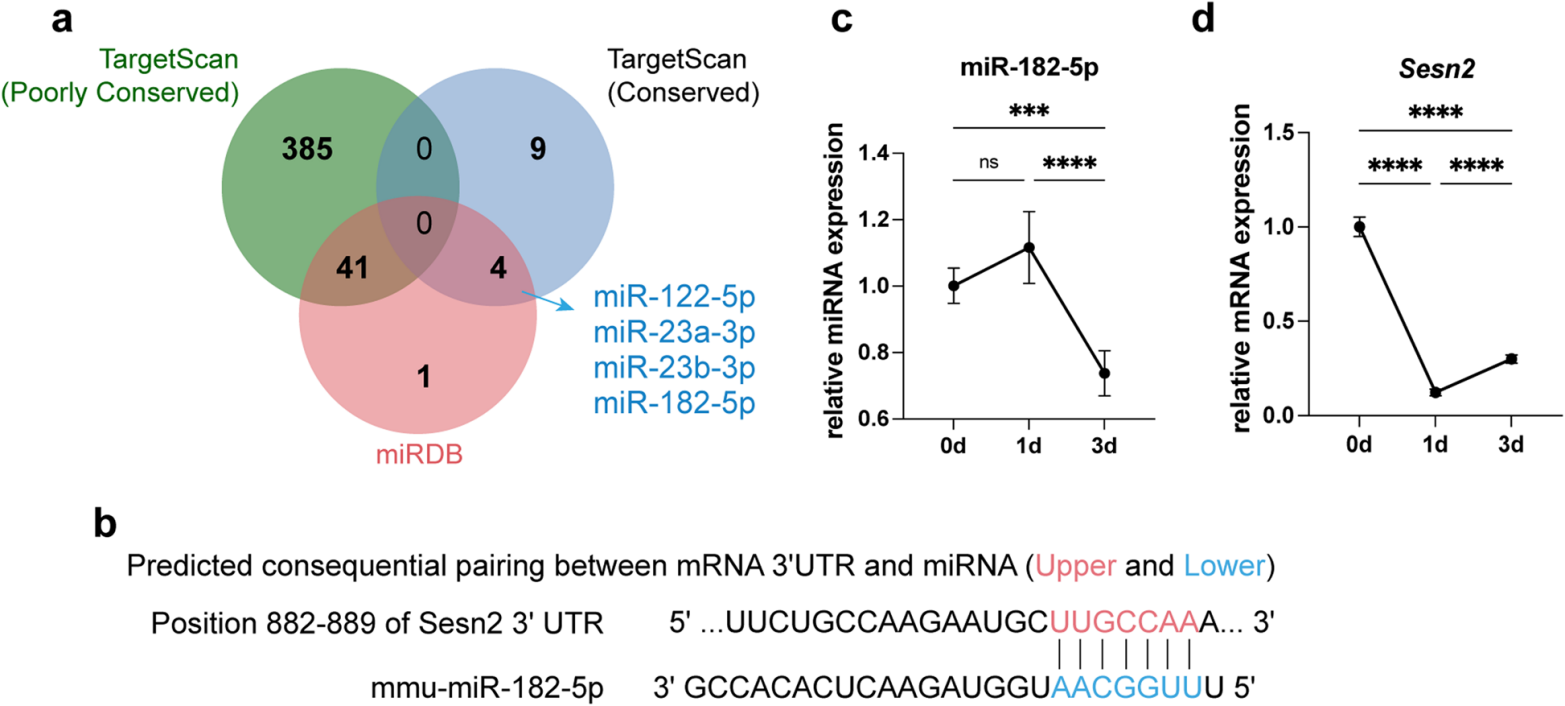
miR-182-5p was identified as a potential regulator of Sesn2. **a** Prediction of miRNAs. Based on the predictions from two databases (TargetScan and miRDB), 4 miRNAs were identified as potentially interacting with *Sesn2* through complementary base pairing. **b** Schematic diagram showing the binding of miRNA-182-5p to the *Sesn2* 3’UTR. **c**, **d** After prolonged induction of differentiation in C2C12 myoblasts, the trends in the expression of miRNA-182-5p and *Sesn2* were examined. One-way ANOVA test. *n* = 6. The data are displayed as the mean $\pm$ standard deviation. ns *p* > 0.05, ***p < 0.001, ****p < 0.0001

*S*ubsequently, we aimed to further study the upstream regulatory mechanisms of Sesn2 during myogenesis. Given the regulatory role of miRNAs in governing gene translation and their potential impact on C2C12 myoblast differentiation [26], we utilized databases to predict miRNAs capable of binding to Sesn2. As shown in Fig. 2a, 46 miRNAs capable of binding to Sesn2 were predicted by the miRDB, while two sets of predictions, namely, a conserved set (containing 13 miRNAs) and a poorly conserved set (containing 426 miRNAs), were obtained via TargetScan. The intersection of the sets of predictions from both databases revealed 4 miRNAs with the potential to bind to *Sesn2* (Fig. 2a). Importantly, complementary binding sites for miR-182-5p were found within a segment of the *Sesn2* 3’UTR (Fig. 2b). The potential interaction between miR-182-5p and *Sesn2* was then investigated further. As the differentiation time increased, the miR-182-5p level initially increased and then decreased (Fig. 2c). Similar to the protein expression trend mentioned above (Figs. 1b and S1c), *Sesn2* mRNA expression decreased on the first day of differentiation and significantly increased on the third day (Fig. 2d). The trends in *Sesn2* and miR-182-5p expression were opposite, indicating the possibility that these two factors interact.

### Sesn2 is regulated by miR-182-5p

**Fig. 3:**
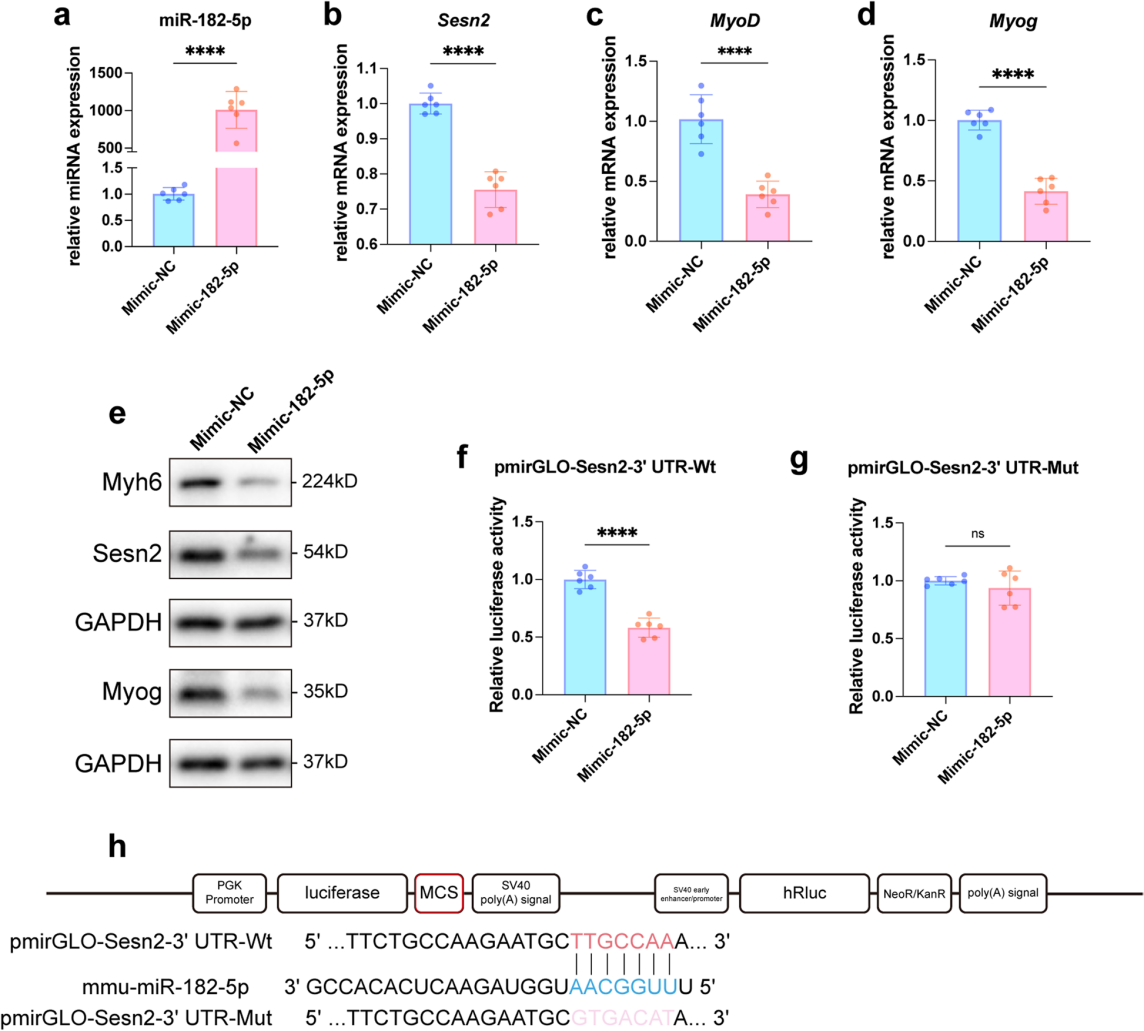
Sesn2 is regulated by miR-182-5p. **a-d** miRNA-182-5p was significantly upregulated in C2C12 myoblasts in the Mimic-182-5p group. Moreover, the mRNA levels of *Sesn2*, *MyoD*, and *Myog* were significantly reduced in the Mimic-182-5p group compared to the Mimic-NC group. Student’s t test. *n* = 6. **e** Measurement of protein expression levels. The protein levels of Sesn2, Myh6, and Myog were significantly lower in the Mimic-182-5p group than those in the control group. **f**, **g** In the dual-luciferase reporter assay, luciferase activity was significantly lower in the pmirGLO-Sesn2-3’UTR-Wt + Mimic-182-5p group than in the pmirGLO-Sesn2-3’UTR-Wt + Mimic-NC group but did not differ significantly between the pmirGLO-Sesn2-3’UTR-Mut + Mimic-182-5p group and pmirGLO-Sesn2-3’UTR-Mut + Mimic-NC group. Student’s t test. *n* = 6. h. Construction of pmirGLO-Sesn2-3’UTR-Wt and pmirGLO-Sesn2-3’UTR-Mut. The data are displayed as the mean $\pm$ standard deviation. *n* = 6. ns *p* > 0.05, ***p < 0.001, ****p < 0.0001

Next, to further confirm the relationship between miR-182-5p and Sesn2, C2C12 myoblasts were divided into two groups: the Mimic-182-5p group (transfected with the miRNA-182-5p mimic) and the Mimic-NC group (transfected with the negative control miRNA). Following transfection, the cells were induced to differentiate for 3 days and were then harvested for subsequent assessment. RT‒qPCR revealed that the relative expression level of miR-182-5p in the Mimic-182-5p group was increased by approximately 1008-fold compared with that in the Mimic-NC group (*p* < 0.0001, Fig. 3a), indicating that miR-182-5p was successfully overexpressed in the Mimic-182-5p group. However, the mRNA level of *Sesn2* in the Mimic-182-5p group decreased by 24.45% compared with that in the Mimic-NC group (*p* < 0.0001, Fig. 3b). Additionally, compared with those in the Mimic-NC group, the *MyoD* and *Myog* mRNA levels in the Mimic-182-5p group were significantly decreased, by 58.63% (*p* < 0.0001, Fig. 3c) and 61.60% (*p* < 0.0001, Fig. 3d), respectively. In further validation of these results, protein expression analysis confirmed that the Sesn2 protein level was significantly lower in the Mimic-182-5p group than in the Mimic-NC group (0.78 $\pm$ 0.06 vs. 1.07 $\pm$ 0.13, *p* < 0.001; Figs. 3e and S1h). The protein expression levels of Myh6 and Myog in the Mimic-182-5p group were also significantly lower than those in the Mimic-NC group, by 64.28% (*p* < 0.001, Figs. 3e and S1i) and 57.84% (*p* < 0.0001, Figs. 3e and S1j), respectively. These results indicate that overexpressing miR-182-5p in C2C12 myoblasts can effectively inhibit Sesn2 expression and myogenesis.

We also constructed two vectors based on the pmirGLO vector: pmirGLO-Sesn2-3’UTR-Wt and pmirGLO-Sesn2-3’UTR-Mut, encoding the wild-type and mutant versions, respectively, of the *Sesn2* 3’UTR sequence containing the miRNA-182-5p binding site (Fig. 3h). These vectors were cotransfected with Mimic-182-5p or Mimic-NC into HEK293T cells. Luciferase activity was significantly lower in the pmirGLO-Sesn2-3’UTR-Wt + Mimic-182-5p group than in the pmirGLO-Sesn2-3’UTR-Wt + Mimic-NC group (Fig. 3f). Moreover, luciferase activity did not differ significantly between the pmirGLO-Sesn2-3’UTR-Mut + Mimic-182-5p and pmirGLO-Sesn2-3’UTR-Mut + Mimic-NC groups (Fig. 3g). These results strongly support the hypothesis that miRNA-182-5p binds to *Sesn2* mRNA.

### Sesn2 is upregulated in the skeletal muscle of mdx mice

**Fig. 4:**
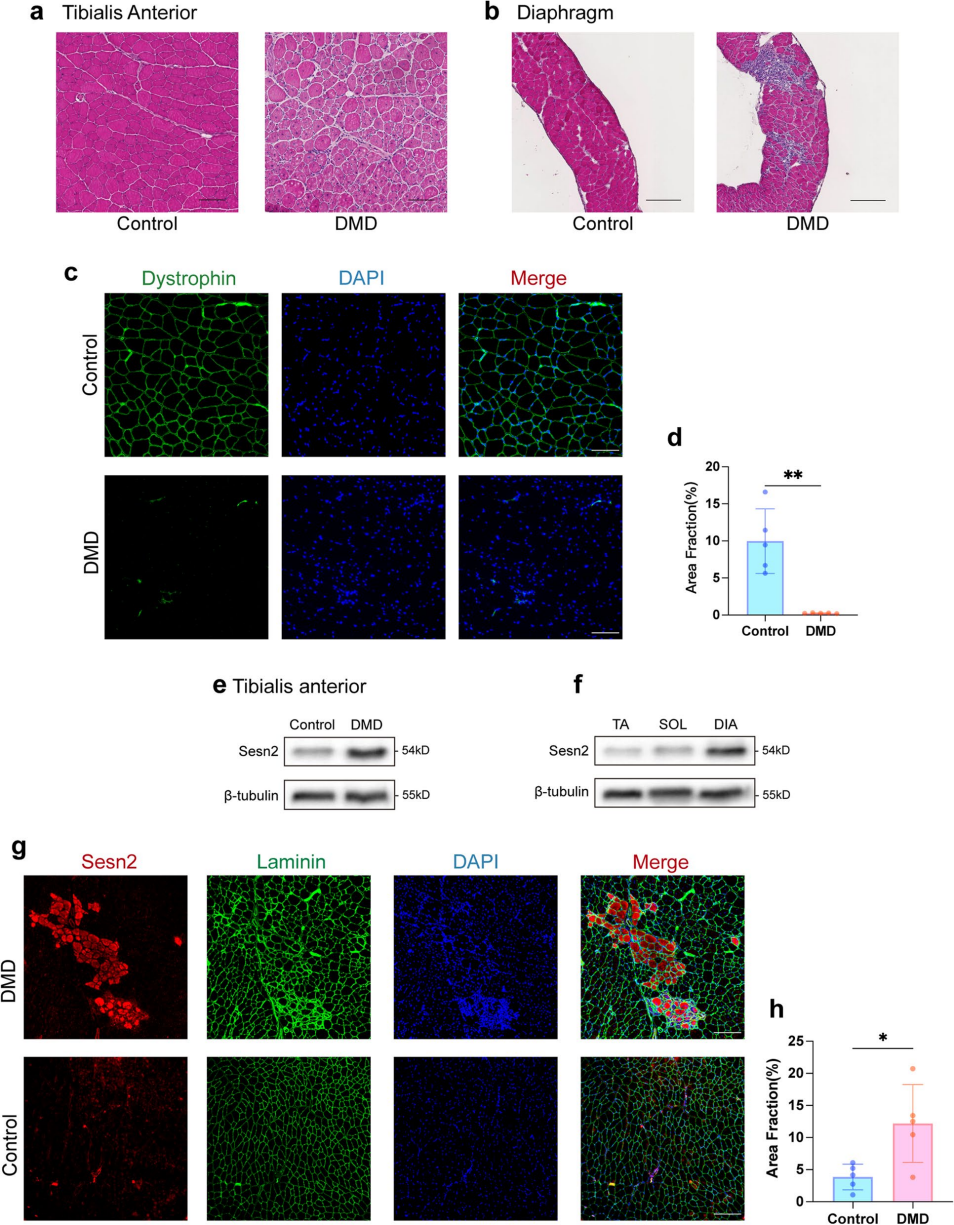
Comparison of Sesn2 expression between the mdx mice and C57BL/10 J mice. **a** H&E staining of the tibialis anterior muscle (TA). Left side, control group; right side, DMD group. Scale bar = 200μm. **b** H&E staining of the diaphragm muscle (DIA). Left side, control group; right side, DMD group. Scale bar = 200μm. **c**, **d** IF analysis of dystrophin in the gastrocnemius muscle (GAS). Clear dystrophin fluorescence was observed in the control group, while the signal was minimal in the DMD group. Scale bar = 100μm. *n* = 5. Student’s t test. **e** Protein expression analysis of Sesn2 in the TA. Sesn2 was significantly upregulated in the DMD group. **f** Comparison of Sesn2 protein levels in the TA, SOL and DIA of mdx mice. The highest expression level was detected in the DIA. **g**, **h** IF detection of Sesn2 levels and distribution in the TA. A notable increase in expression was observed in the DMD group. Scale bar = 200μm. Student’s t test. The data are displayed as the mean $\pm$ standard deviation. *n* = 5. Student’s t test. *p < 0.05, **p < 0.01

Above results revealed that inhibition of Sesn2 suppressed myogenic differentiation of C2C12 myoblasts, suggesting a potential role for Sesn2 in skeletal muscle regeneration. To further verify the biological function of Sesn2, we selected mdx mice for further in-depth study. The mdx mouse is a classic animal model for DMD characterized by the coexistence of necrosis and regeneration processes in its skeletal muscles. Comparing to those in C57BL/10 J mice (control group), hematoxylin and eosin staining (H&E) staining revealed significant inflammatory cell infiltration in the tibialis anterior muscle (TA) and diaphragm muscles (DIA) in the mdx mice (DMD group), along with a considerable number of centrally nucleated myofibers (Fig. 4a and b). Inflammatory cell infiltration was more pronounced in the DIA of mdx mice (Fig. 4b). Some myofibers even contained multiple centrally localized nuclei. These phenomena indicate the occurrence of damage and self-repair processes in skeletal muscle in the DMD model. Additionally, IF staining of the gastrocnemius muscle (GAS) revealed the presence of dystrophin on the sarcolemma in the control group (Fig. 4c). In comparison, the DMD group exhibited a marked reduction in the fluorescence signal of dystrophin, approaching 0% (*p* < 0.01, Fig. 4c and d). This finding suggested the absence of the dystrophin protein on the sarcolemma in the DMD group, consistent with the genetic and molecular characteristics of DMD.

Next, we examined Sesn2 expression in the skeletal muscle of the DMD group compared with the control group. WB analysis revealed significant upregulation of Sesn2 in the TA, DIA and GAS of the DMD group compared to the control group (Figs. 4e and S2a-S2c). In the GAS, Sesn2 expression in the DMD group was increased by 84.85% compared with that in the control group (0.61 $\pm$ 0.18 vs. 0.33 $\pm$ 0.19, *p* < 0.05; Fig. S2a). In the TA, the relative expression level of Sesn2 in the DMD group was increased by 72.09% compared with that in the control group (0.74 $\pm$ 0.09 vs. 0.43 $\pm$ 0.11, *p* < 0.01; Figs. 4e and S2c). DIA revealed that the relative expression level of Sesn2 in the DMD group was 54.55% greater than that in the control group (0.68 $\pm$ 0.07 vs. 0.44 $\pm$ 0.14, *p* < 0.05; Fig. S2b). Furthermore, considering the degrees of fibrosis and inflammation reported in the literature [27], the DIA was found to be the most severely affected, followed by the TA and the soleus muscle (SOL). WB analysis revealed that Sesn2 expression was greater in the DIA than those in either the TA or the SOL (Figs. 4f and S2d). These findings suggested that Sesn2 expression is elevated in the regions with the most severe damage. Subsequent IF analysis of the distribution and relative expression of Sesn2 further confirmed the significant increase in Sesn2 protein levels in the DMD group (Fig. 4g and h), indicating a potential association between Sesn2 and disease progression in mdx mice.

### Sesn2 is associated with the degree of skeletal muscle regeneration in mdx mice

**Fig. 5:**
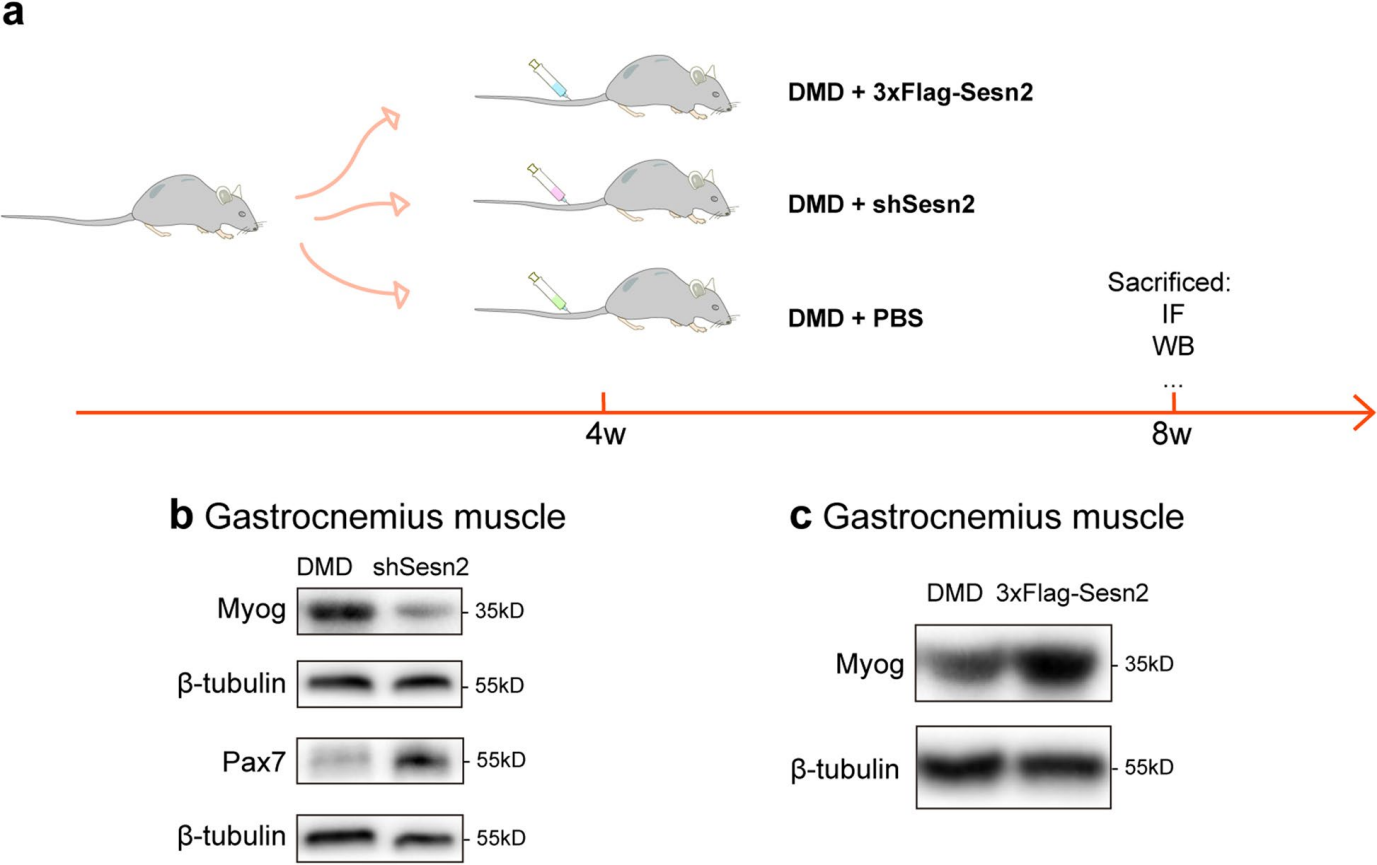
Influence of Sesn2 on the expression of myogenic regulatory factors in the skeletal muscles of mdx mice. **a** Schematic diagram of the mouse modeling process. Four-week-old mdx mice were divided into three groups: the overexpression group (3xFlag-Sesn2 group), in which the mice were administered AAV (AAV2/9-CMV-3xflag-Sesn2-ZsGreen) overexpressing Sesn2; the knockdown group (shSesn2 group), in which the mice were administered AAV2/9-U6-Sesn2-shRNA-EGFP targeting Sesn2 for knockdown; and the control group (DMD group), in which the mice were administered an equivalent volume of PBS. All treatments were administered via tail vein injection. Tissues were collected one month postinjection for subsequent experimental assessments. **b** Protein levels of Myog and Pax7 in the GAS. There were significant decreases in the protein expression levels of Myog in the shSesn2 group. Pax7 expression was significantly increased in the shSesn2 group. **c** Protein levels of Myog in the GAS. In the 3xFlag-Sesn2 group, Myog expression was significantly upregulated

Given the notable upregulation of Sesn2 in the skeletal muscle of mdx mice, we sought to further study the impact of Sesn2 on mdx mouse skeletal muscle by establishing models of Sesn2 overexpression or knockdown. The mdx mice were divided into 3 groups. At 4 weeks of age, the mice received an intravenous (tail vein) injection of AAV to induce Sesn2 overexpression (3xFlag-Sesn2 group) or knockdown (shSesn2 group) in skeletal muscle. The control group of mdx mice (DMD group) was administered an equivalent volume of PBS. Tissue samples were harvested one month postinjection for subsequent experiments (Fig. 5a). The expression of Sesn2 was validated by WB analysis. In the TA, Sesn2 expression was decreased by 50.68% in the shSesn2 group compared with that in the DMD group (*p* < 0.05, Fig. S3a and S3b), while in the GAS, Sesn2 expression was decreased by 45.45% (*p* < 0.05, Fig. S3c and S3d). Conversely, the relative expression level of Sesn2 in the 3xFlag-Sesn2 group was significantly greater than that in the DMD group in both the TA (0.80 $\pm$ 0.06 vs. 0.02 $\pm$ 0.01, *p* < 0.0001; Fig. S3e and S3f) and GAS (0.84 $\pm$ 0.25 vs. 0.02 $\pm$ 0.01, *p* < 0.05; Fig. S3g and S3h). These results validated the successful overexpression and knockdown of Sesn2.

Then, we focused on the influence of Sesn2 overexpression and knockdown on skeletal muscle regeneration in mdx mice. Given that the compromised myogenic differentiation contributes to impaired skeletal muscle regeneration in DMD, we investigated the changes in the expression of myogenic regulatory factors (MRFs). Compared with the DMD group, the shSesn2 group exhibited a 63.93% decrease in Myog expression in the TA (0.22 $\pm$ 0.10 vs. 0.61 $\pm$ 0.23, *p* < 0.05; Fig. S3i and S3j). In the GAS, Myog expression in the shSesn2 group was also significantly lower than that in the control group (0.38 $\pm$ 0.09 vs. 0.79 $\pm$ 0.20, *p* < 0.05; Figs. 5b and S3o). Compared with that in the DMD group, the expression of desmin in the GAS was decreased by 57.69% in the shSesn2 group (0.33 $\pm$ 0.08 vs. 0.78 $\pm$ 0.18, *p* < 0.01; Fig. S3k and S3l). However, examination of Pax7 (a marker of satellite cells) in the GAS revealed that the expression level in the shSesn2 group was significantly greater than that in the DMD group (0.85 $\pm$ 0.27 vs. 0.29 $\pm$ 0.06, *p* < 0.01; Figs. 5b and S3q). Conversely, in the TA, the relative expression level of Myog in the 3xFlag-Sesn2 group was increased by 1.41-fold compared with that in the DMD group (1.81 $\pm$ 0.19 vs. 0.75 $\pm$ 0.36, *p* < 0.01; Fig. S3m and S3n). Furthermore, in the GAS, the relative expression levels of Myog (1.34 $\pm$ 0.17 vs. 0.86 $\pm$ 0.11, *p* < 0.01) in the 3xFlag-Sesn2 group were significantly increased compared with those in the DMD group (Figs. 5c and S3p). Thus, the findings of the present study suggested that Sesn2 is involved in the process of skeletal muscle regeneration in mdx mice.

### Overexpression of Sesn2 elevates the proportion of slow-twitch myofibers in the skeletal muscle of mdx mice

**Fig. 6:**
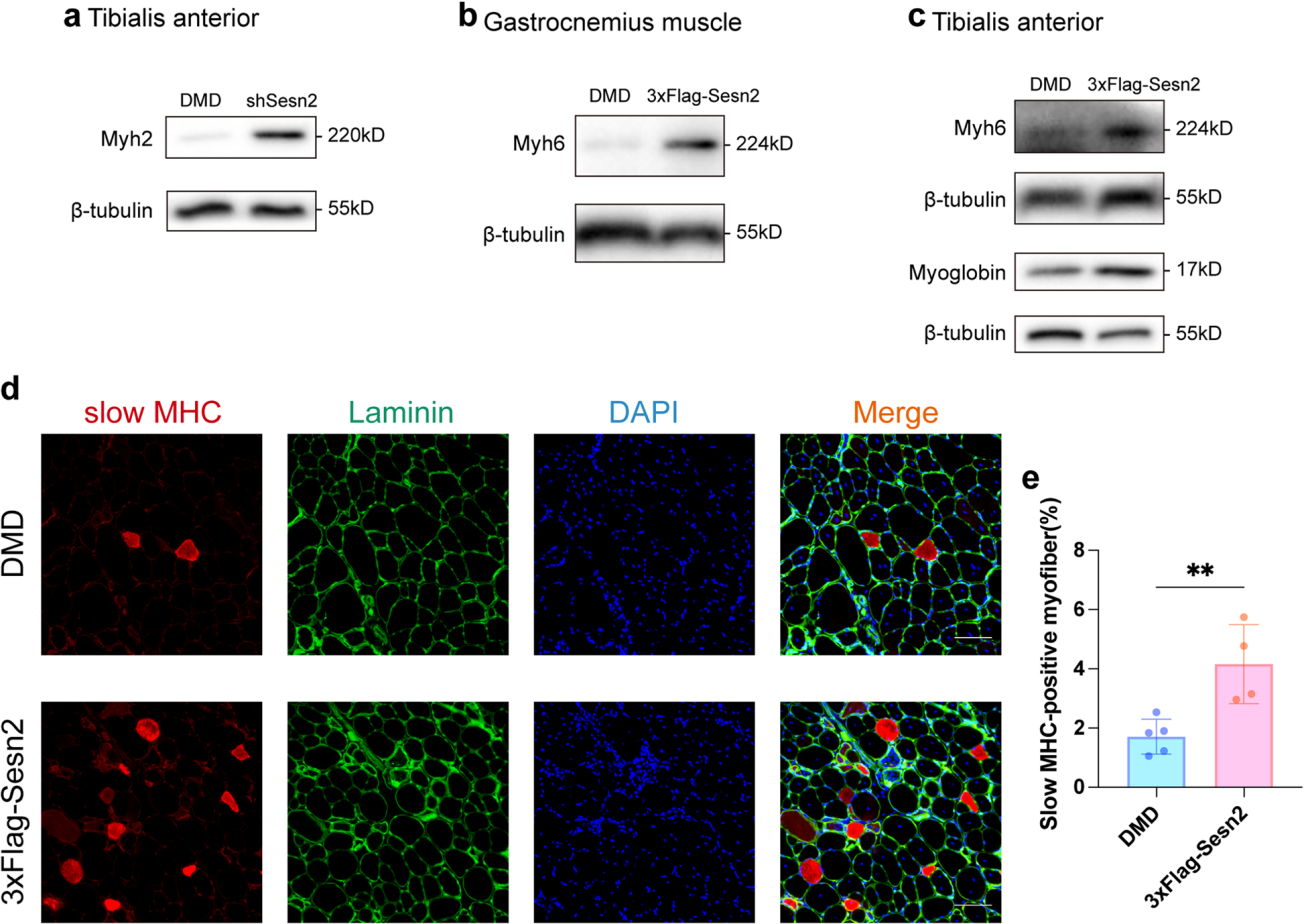
Effect of Sesn2 on the proportion of slow-twitch myofibers in the skeletal muscle of mdx mice. **a** Impact of Sesn2 knockdown on Myh2 expression in the TA. In the shSesn2 group, a pronounced increase in Myh2 expression was observed in the TA. **b** Upregulation of Myh6 in the GAS following Sesn2 overexpression. **c** Increased Myh6 and myoglobin levels in the TA of mice in the 3xFlag-Sesn2 group. **d**, **e** Increased proportion of slow MHC-positive myofibers in the 3xFlag-Sesn2 group. Student’s t test. Scale bar = 100μm. The data are displayed as the mean $\pm$ standard deviation. *n* = 4–5. *p < 0.05, **p < 0.01

Given the above findings indicating that the modulation of Sesn2 expression can influence the expression of Myh6 (a slow-twitch myofiber marker), and the preliminary evidence suggesting the effect of Sesn2 on the slow-to-fast myofiber transition [3], we next explored the impact of Sesn2 on the myofiber type in mdx mice. WB analysis revealed a significant increase in the relative protein level of the fast-twitch myofiber marker Myh2 in the shSesn2 group compared to the DMD group (0.51 $\pm$ 0.33 vs. 0.09 $\pm$ 0.06, *p* < 0.05; Figs. 6a and S4a). In the 3xFlag-Sesn2 group, overexpression of Sesn2 resulted in a significant increase in the protein expression levels of slow-twitch myofiber markers. Specifically, the expression of Myh6 in the GAS and TA was increased by approximately 2.4-fold (*p* < 0.05, Figs. 6b and S4b) and 1.83-fold (*p* < 0.05, Figs. 6c and S4c), respectively, in the 3xFlag-Sesn2 group. Notably, the relative protein expression of myoglobin in the 3xFlag-Sesn2 group was increased by 63.33% compared with that in the DMD group (*p* < 0.01, Figs. 6c and S4d). Moreover, IF analysis revealed a substantial increase in the proportion of slow MHC-positive myofibers in the TA following Sesn2 overexpression (*p* < 0.01, Fig. 6d and e). These data indicate that Sesn2 influences the myofiber type transition in mdx mice; specifically, Sesn2 overexpression increases the proportion of slow-twitch myofibers.

## Discussion

Findings from earlier studies suggested a connection between Sesn2 and skeletal muscle atrophy [3], which is a pathological condition related to the regenerative capacity. Specifically, the impaired myogenic differentiation capacity of SCs results in diminished regenerative capacity [28]. Therefore, understanding the factors governing myogenic differentiation is significant for developing therapies for muscular disorders.

A previous review [21] has inferred that Sesn2 could regulate myogenic differentiation of C2C12 myoblasts by affecting mitophagy, but there is currently no direct evidence to confirm this relationship. To investigate the influence of Sesn2, we first examined the expression trend of Sesn2 over the course of C2C12 myoblasts differentiation. The results indicated that both the protein and mRNA expression levels first decreased and then increased. Earlier study [29] have shown that the mRNA expression level of Sesn2 decreases after 3 days of C2C12 myoblasts differentiation, but they did not conduct further analysis at the protein expression level. The initial decrease in expression during the early stages of differentiation may be a component of the regulatory mechanism that primes the cells for differentiation. The subsequent gradual increase in the Sesn2 expression as differentiation advances suggests that elevated levels of Sesn2 are necessary for the later phases of myogenic differentiation, such as myotube formation. Moreover, further validation revealed that knocking down the expression of Sesn2 leads to a reduced differentiation capacity of C2C12 myoblasts, as evidenced by impaired myotube formation and reduced levels of MRFs such as Myog. These results strongly indicate that the Sesn2 is essential for proper myoblasts differentiation into myotubes, which aligns with previous hypothesis [21]. However, we did not investigate changes in autophagy or mitophagy. This represents a limitation of our current research. More studies should aim to elucidate the role of autophagy and mitophagy in this context by measuring changes in autophagic and mitophagic activity following the inhibition of Sesn2. For instance, it is necessary to assess the levels of key autophagy markers such as LC3B and p62 [30] at first.

Earlier studies [22, 23] indicated that miRNAs could influence the myogenic differentiation of C2C12 myoblasts. In this study, we performed bioinformatic analysis to identify 4 potential miRNAs which may regulate the activity of Sesn2. A previous study [26] have confirmed that miR-122-5p can reverse the inhibition of myogenic differentiation in C2C12 cells induced by TGF-$\upbeta$. However, we found that the overexpression of miR-122-5p did not result in any change in the expression of Sesn2 (data not shown). This findings suggested that Sesn2 is likely not regulated by miR-122-5p during the differentiation of C2C12 myoblasts. Next, we analyzed the changes in the expression of miR-182-5p. RT-qPCR analysis revealed that the changes in the mRNA expression of miR-182-5p was opposite to that of Sesn2. Considering the mechanism by which miRNAs bind to target mRNAs to cause degradation or translation inhibition [24], these results suggested a potential interaction between them. Further validation revealed that overexpression of miR-182-5p in C2C12 myoblasts led to a significant decrease in both the protein and mRNA expression levels of Sesn2, concomitant with impaired myogenic differentiation. This inhibitory effect of miR-182-5p on Sesn2 expression and subsequent myogenesis highlights the significance of this miRNA-mediated regulatory axis in governing the myogenic program. This study could pave the way for developing targeted therapeutic strategies by regulating the miR-182-5p-Sesn2 axis to promote the function of SCs and improve regeneration in the context of muscular dystrophy, such as DMD.

To substantiate these in vitro findings, we investigated the role of Sesn2 in the skeletal muscle of a mdx mouse model of DMD. H&E staining revealed that centrally nucleated myofibers and inflammatory cells infiltration coexisted in the skeletal muscle of mdx mice. These results highlight the ongoing cycles of muscle degeneration and regeneration, which are consistent with previous studies [31]. As regeneration is closely associated with myogenic differentiation of SCs, the mdx mouse is suitable for investigating the role of Sesn2 on regenerative capacity of skeletal muscle. Our further findings, obtained from WB and IF analysis, demonstrated an upregulation of Sesn2 in the skeletal muscles of mdx mice. This observation is similar to that of an earlier study [3] that reported an increased expression of Sesn2 in a denervated muscle atrophy model. Inspired by the results of a previous study [3], upregulation of Sesn2 may play a protective role in promoting regeneration of the skeletal muscles of mdx mice.

To verify this hypothesis, we generated the Sesn2-overexpression and Sesn2-knockdown mdx mice. Subsequent protein analysis revealed that knockdown of Sesn2 in the skeletal muscle of mdx mice resulted in decreased expression of Myog, a key MRF for terminal differentiation of SCs [32], and increased expression of Pax7, a marker for quiescent SCs [7, 8]. Conversely, overexpression of Sesn2 led to an increase in Myog expression levels. This findings suggested that Sesn2 positively regulates myogenesis by promoting the expression of Myog, thereby driving myoblasts myogenic differentiation and increasing the regenerative degree of regeneration. Upregulation of Pax7 expression following Sesn2 knockdown may indicate a shift toward maintaining a more quiescent state, potentially hindering effective muscle regeneration. In contrast, the upregulation of Myog upon Sesn2 overexpression supports the notion that Sesn2 facilitates myogenic differentiation, aiding in regeneration. However, this study did not directly test the expression of embryonic myosin heavy chain (eMyHC), which is related to newly formed myofibers during regeneration [33]. Collectively, these findings suggest that Sesn2 plays a pivotal role in regulating the myogenic program in mdx mice.

Early experiments [3] confirmed that knocking down Sesn2 in a denervation-induced atrophy model accelerates the progression of skeletal muscle atrophy and is induced by the conversion of slow-twitch to fast-twitch myofibers. In our study on C2C12 myoblasts, after Sesn2 expression was knocked down, the Myh6 expression level decreased significantly. Moreover, our study showed that Sesn2 knockdown in the mdx mice resulted in significantly increased expression of fast-twitch myosin heavy chain 2 (Myh2 [34]), while Sesn2 overexpression led to significantly elevated levels of the slow-twitch myofiber markers Myh6 [35] and Myoglobin [3] and an increased proportion of slow-twitch myofibers. These findings parallel those of previous research [3] suggesting the important role of Sesn2 in elevating the proportion of slow-twitch myofibers. Considering the crucial role of slow-twitch myofibers in fatigue resistance and exercise endurance, previous literature [36] has indicated that increasing the number of slow-twitch myofibers could be a therapeutic strategy for attenuating DMD phenotypes. Our findings suggest that interventions targeting Sesn2 could increase the proportion of slow-twitch myofibers in mdx mice. However, this study is a preliminary study on the effect of Sesn2 on slow-twitch myofibers in the skeletal muscle of mdx mice, and the specific mechanism through which Sesn2 causes this elevation in this model is worthy of further study.

In summary, our research revealed Sesn2 as a novel regulator of myogenic differentiation that acts downstream of miR-182-5p. Sesn2 overexpression increases the proportion of slow-twitch myofibers and increased the degree of regeneration in the skeletal muscle of mdx mice. These findings provide a potential therapeutic target for enhancing the regenerative capacity of skeletal muscle and alleviating muscular wasting conditions associated with impaired myogenesis.

## Materials and methods

### Recombinant Adeno-associated Virus (AAV)

To achieve Sesn2 overexpression or knockdown in mouse skeletal muscle, AAVs were generated. AAV2/9-CMV-Sesn2-3xflag-ZsGreen (titer: 1.5 $\times$ 10^12^vg/ml) was engineered to selectively overexpress Sesn2, and AAV2/9-U6-Sesn2-shRNA-EGFP (titer: 1.2 $\times$ 10^12^ vg/ml) was developed to specifically express the shRNA sequence for Sesn2 knockdown. The Sesn2 shRNA sequence in AAV2/9-U6-Sesn2-shRNA-EGFP was 5’-GCAUCAGAUACGAUGACUA-3’. Both viruses were produced by Hanbio (Shanghai, China).

### Animals

Male C57BL/10 J mice and DMD model mice (mdx mice; C57BL/10ScSnJGpt-Dmd em3Cd4/Gpt) were purchased from GemPharmatech Biotechnology. Four-week-old male mdx mice were divided into three groups: the mdx mice + AAV2/9-CMV-Sesn2-3xflag-ZsGreen group (3xFlag-Sesn2 group), the mdx mice + AAV2/9-U6-Sesn2-shRNA-EGFP group (shSesn2 group) and the mdx mice + PBS group (DMD group). Each mdx mouse received an injection of 120μl AAV, and observations and assessments were conducted one month post-injection. The experimental mice were housed in controlled environments, with a 12-h light/dark cycle, a temperature of 22 °C, and 50–60% humidity. The animals were provided free access to both bacteria-free water and standard laboratory food. The protocols for animal experiments were reviewed and approved by the Institutional Animal Care and Use Committee of Sun Yat-Sen University (SYSU-IACUC-2023–001741).

### Cell culture

C2C12 myoblasts cells were obtained from the Cell Bank of the Chinese Academy of Sciences. For proliferation and passaging, C2C12 myoblasts were cultured in growth medium (GM) comprising Dulbecco’s modified Eagle’s medium (DMEM; Gibco, C11995500BT) supplemented with 10% fetal bovine serum (FBS; Gibco, 10,091–148) in a humidified atmosphere with 5% CO2 at 37 °C. To induce C2C12 myoblast differentiation, the GM was replaced with differentiation medium (DM) consisting of DMEM and 2% horse serum (HS; Gibco, 26,050,088).

### Cell transfection

To investigate the role of Sesn2 in C2C12 myoblast differentiation, cells were transfected with a specific siRNA to knock down Sesn2 using Lipofectamine 3000 (Lipo3000; Gibco, L3000015) according to the manufacturer’s instructions. Before transfection, the cells were seeded in a 6-well plate and allowed to grow to 70–80% confluence.

First, the siRNA and Lipo3000 were solubilized in two EP tubes, each containing 125μl Opti-MEM. Then, the diluted siRNA was transferred to an EP tube containing Lipo3000 and incubated for 15 min at room temperature. Finally, the siRNA and Lipo3000 complexes were added to each well and incubated for 12 h. The transfected cells were then used in experiments. The miR-182-5p mimic transfection procedure was similar to the siRNA transfection procedure detailed above. The Sesn2 siRNA sense sequence was 5’-GCAUCAGAUACGAUGACUA-3’, and the miR-182-5p mimic sequence was 5’- UUUGGCAAUGGUAGAACUCACACCG-3’. The siRNA, negative control siRNA (siNC), miR-182-5p mimic (Mimic-182-5p) and miRNA mimic negative control (Mimic-NC) were synthesized by and obtained from Tsingke Biological Technology (Beijing, China).

For plasmid transfection, 2μg of plasmid was mixed with 4μl of P3000 (provided in the kit) in an EP tube, to which Lipo3000 was then added. After incubation for 15 min, the mixture was added to the medium.

### RNA extraction and real-time quantitative polymerase chain reaction (RT-qPCR)

In accordance with the manufacturer’s instructions, AG RNAex Pro Reagent (Accurate Biotechnology, AG21102) was used to extract total RNA from cells. The RNA quality and concentration were determined by a NanoDrop2000 spectrophotometer (Thermo Scientific). Then, 1μg of total RNA was reverse transcribed into cDNA using an Evo M-MLV RT Reaction Mix Kit (Accurate Biotechnology, AG11728). For miRNA analysis, 0.5μg total RNA was reverse transcribed using an Evo M-MLV RT Kit for qPCR (Accurate Biotechnology, AG11707). RT-qPCR was performed using a SYBR Green Premix Pro Taq HS qPCR Kit (Accurate Biotechnology, AG11719) according to the manufacturer’s instructions. The internal controls were GAPDH (for mRNA) and U6 (for miRNA). The primer sequences are provided in supplementary Table S1.

### Western Blot (WB) analysis

Total protein was extracted from the cells or skeletal muscle samples by lysis in RIPA buffer (Thermo Scientific, 89,900) containing 1 mM PMSF (Beyotime, ST506) and PhosSTOP (Roche, 4,906,845,001). The protein concentration in each sample was detected with a Pierce BCA assay kit (Thermo Scientific, 23,227) following the manufacturer’s instructions. Then, 10% or 12.5% SDS–polyacrylamide gel electrophoresis (SDS-PAGE) was used to separate equal amounts of proteins. After separation, the proteins were transferred to 6.5 $\times$ 7 or 8.5 $\times$ 7 mm PVDF membranes (Millipore), which were then blocked with 5% BSA in TBST (500 mM NaCl, 20 mM Tris–HCl, 0.2% Tween) for 1 h and incubated overnight at 4 °C with primary antibodies against the following proteins: Myoglobin (Abcam, ab77232), Myh2 (Abcam, ab124937), Myh4 (Proteintech, 20,140–1-AP), Myh6 (Abcam, ab185967), Sesn2 (Abcam, ab178518), Myog (Abcam, ab124800), Desmin (Abcam, ab32362), Pax7 (DSHB). Finally, the membranes were incubated for 1 h with HRP-conjugated anti-rabbit IgG antibodies (CST, 7074S) or HRP-conjugated anti-mouse IgG antibodies (CST, 7076S). The proteins were visualized with a chemiluminescence detection system. GAPDH (CST, 2118S) or β-tubulin (CST, 2128S) was used as the internal reference.

### Immunofluorescence (IF) assay

IF assay was conducted to evaluate the effect of Sesn2 on C2C12 myoblast differentiation. First, the cells were washed twice with phosphate-buffered saline (PBS) and fixed with 4% paraformaldehyde (PFA) for 20 min at room temperature. Then, the cells were blocked for 30 min with QuickBlock™ Blocking Buffer for Immunol Staining (Beyotime, P0260) and then incubated with primary antibody at 4 °C overnight. For IF analysis of skeletal muscle, frozen samples were sectioned at a 10μm thickness, fixed with acetone for 10 min, and washes twice with PBS for 10 min each. Then, the samples were blocked with goat serum for 1 h prior to overnight incubation with primary antibodies against the following proteins: myosin heavy chain, sarcomere (MHC) antibody (DSHB, MF20), dystrophin (Abcam, ab275391), Sesn2 (Proteintech, 10,795–1-AP), laminin (Sigma, L9393 or L8271), and slow MHC (Sigma, M8421). Next, the cells or frozen skeletal muscle sections were washed three times with PBS, and were then incubated with suitable secondary antibodies, including anti-rabbit Alexa Fluor 488-conjugated (CST, 4412S), anti-rabbit Alexa Fluor 555-conjugated (CST, 4413S), anti-rabbit Alexa Fluor 647-conjugated (CST, 4414S), anti-mouse Alexa Fluor 488-conjugated (CST, 4408S), anti-rabbit mouse Fluor 555-conjugated (CST, 4409S) and anti-mouse Alexa Fluor 647-conjugated (CST, 4410S) secondary antibodies. Next, the cells were washed 3 times with PBS and stained with DAPI (Sigma, F6057) to visualize the nuclei. Finally, the cells were observed and photographed under a fluorescence microscope. Multinucleated myotubes were defined as MHC-positive cells with two or more nuclei. The fusion index was calculated by dividing the number of nuclei in each multinucleated myotube by the total number of nuclei.

### Prediction of target miRNAs

To investigate the potential upstream miRNAs regulating Sesn2, the TargetScan and miRDB databases were utilized to predict miRNAs capable of binding to Sesn2. Subsequently, the sets of predicted miRNAs obtained from each database were intersected to narrow the list of target miRNAs.

### Dual-Luciferase reporter assay

To investigate the interaction between Sesn2 and miR-182-5p, the miR-182-5p binding site within the 3’UTR of Sesn2 mRNA, as well as a sequence containing a mutated binding site, was cloned and inserted into the pmirGLO Dual-Luciferase miRNA target expression vector (pmirGLO vector). Two reporter vectors were constructed by Tsingke Biological Technology (Beijing, China): the wild-type reporter vector (pmirGLO-Sesn2-3’UTR-Wt) and the mutant reporter vector (pmirGLO-Sesn2-3’UTR-Mut). HEK-293 T cells were co-transfected with either miR-182-5p mimic or mimic-NC, along with the above reporter vectors, using Lipo3000 as the transfection reagent. After 24-48 h of incubation, luciferase activity was determined utilizing a dual-luciferase reporter assay kit (Promega, E1910).

### Hematoxylin and Eosin staining (H&E staining)

Frozen sections were hydrated by two immersions for 3 min each and were then stained with hematoxylin (Baso, BA4021A) for 2 min. The sections were rinsed twice with water for 3 min each, stained for 1 min with eosin (Beyotime, C0109), and rinsed twice more with water for 3 min each. The sections were dehydrated in an alcohol series (75%, 85%, 95%, 100%, and 100% again). The sections were then immersed twice in xylene for 5 min each. Finally, the sections were sealed with neutral resin and placed under a microscope for observation, and images were captured.

### Statistical analysis

For comparisons between two groups, Student’s t test was used to assess the significant of differences in normally distributed data. For comparisons of nonnormally distributed data, the Mann–Whitney U test was used. For comparisons involving more than two datasets, one-way ANOVA was conducted to assess the significance of differences. A *p* value < 0.05 was considered to indicate statistical significance. All statistical analysis were conducted using GraphPad Prism (version: 9.3.1) The data are presented as the mean $\pm$ standard deviation.

### Supplementary Information

Supplementary Material 1.

## Data Availability

All data presented in this paper could be accessible through a material transfer agreement by contacting the corresponding author.
