# Supplementary material for: Inhibition of Sesn2 has negative regulatory effects on the myogenic differentiation of C2C12 myoblasts
Source: Mol Biomed. 2024 Aug 9;5:31. doi: 10.1186/s43556-024-00193-z (PMC11310181; doi:10.1186/s43556-024-00193-z)
Supplement: Supplementary file 1 — Supplementary Material 1. [file 43556_2024_193_MOESM1_ESM.docx]

**Supplementary information:**

**Inhibition of Sesn2 has negative regulatory effects on the myogenic differentiation of C2C12 myoblasts**

Authors: Zubiao Song^1^, Qing Lin^1^, Jiahui Liang ^1^, Weixi Zhang^1, *^

Address: 1. Department of Neurology, The First Affiliated Hospital, Sun Yat-sen University; Guangdong Provincial Key Laboratory of Diagnosis and Treatment of Major Neurological Diseases; National Key Clinical Department and Key Discipline of Neurology, No.58 Zhongshan Road 2, Guangzhou, 510080, China.

*Correspondence author: Weixi Zhang ([zhangwxi@mail.sysu.edu.cn](mailto:zhangwxi@mail.sysu.edu.cn))


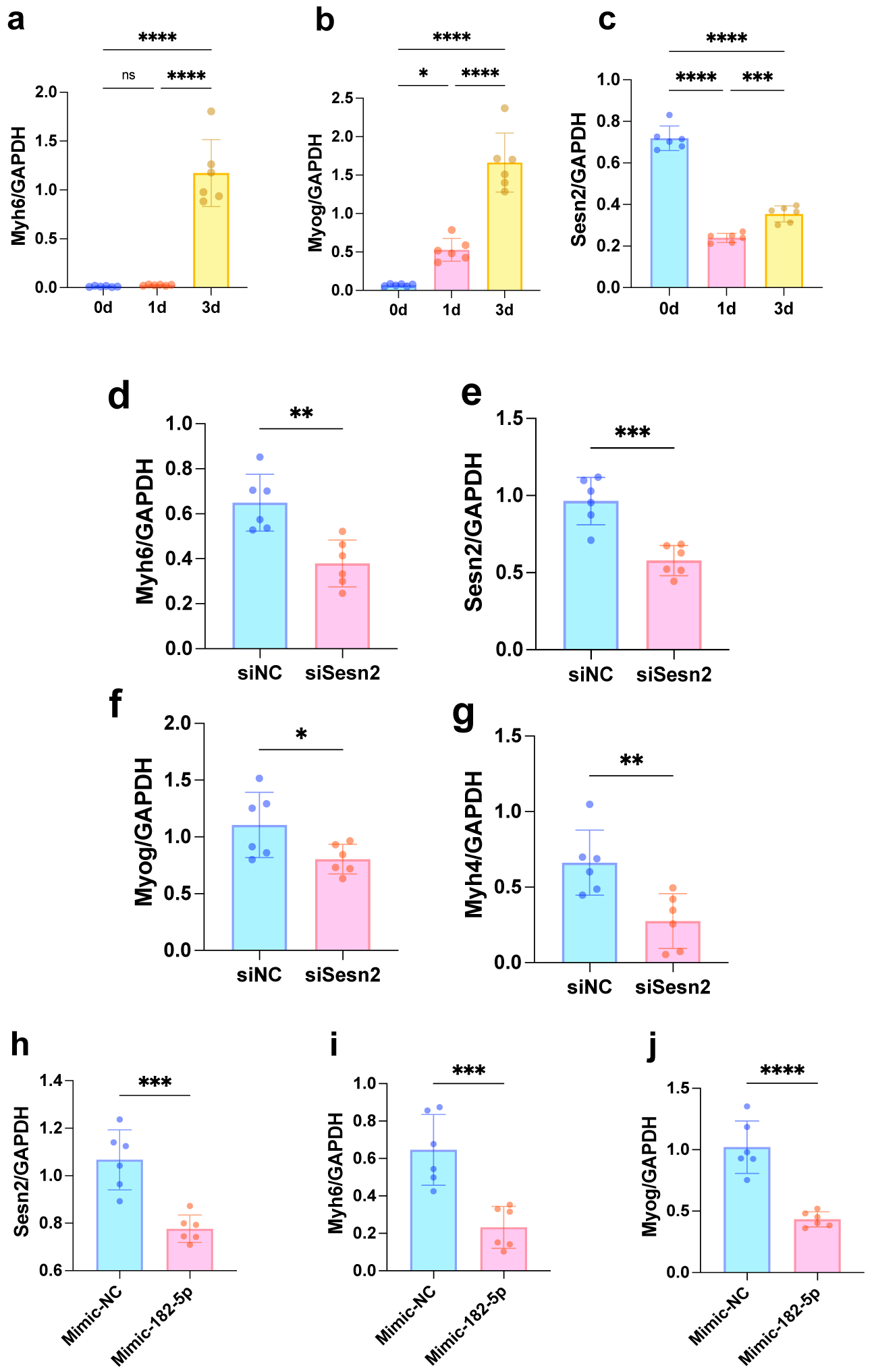


**Fig. S1** Statistical analysis of protein expression levels from western blot assays. a-c. Changes in the expression trend of Myh6, Myog and Sesn2 with the prolongation of the myogenic differentiation time of C2C12 myoblasts. One-way ANOVA test. n=6. d-g. Statistical analysis of the expression trends of Myh6, Myog and Myh4 after knockdown of Sesn2. e. The protein expression level of Sesn2 was significantly knocked down by siRNA. d, f, g. It was evident that the protein expression levels of Myh6, Myog and Myh4 showed a significant decrease. n=6. Student’s t test. h-j. Statistical analysis of the expression trends of Myh6, Myog and Sesn2 after miR-182-5p overexpression. h. Overexpression of miR-182-5p significantly inhibited the protein expression of Sesn2. i, j. Statistical analysis revealed that the protein expression levels of Myh6 and Myog were significantly reduced in the Mimic-182-5p group. n=6. Student’s t test. The data are displayed as the mean $\pm$ standard deviation. ns *p* > 0.05, **p* < 0.05, ***p* < 0.01, ****p* < 0.001, *****p* < 0.0001.


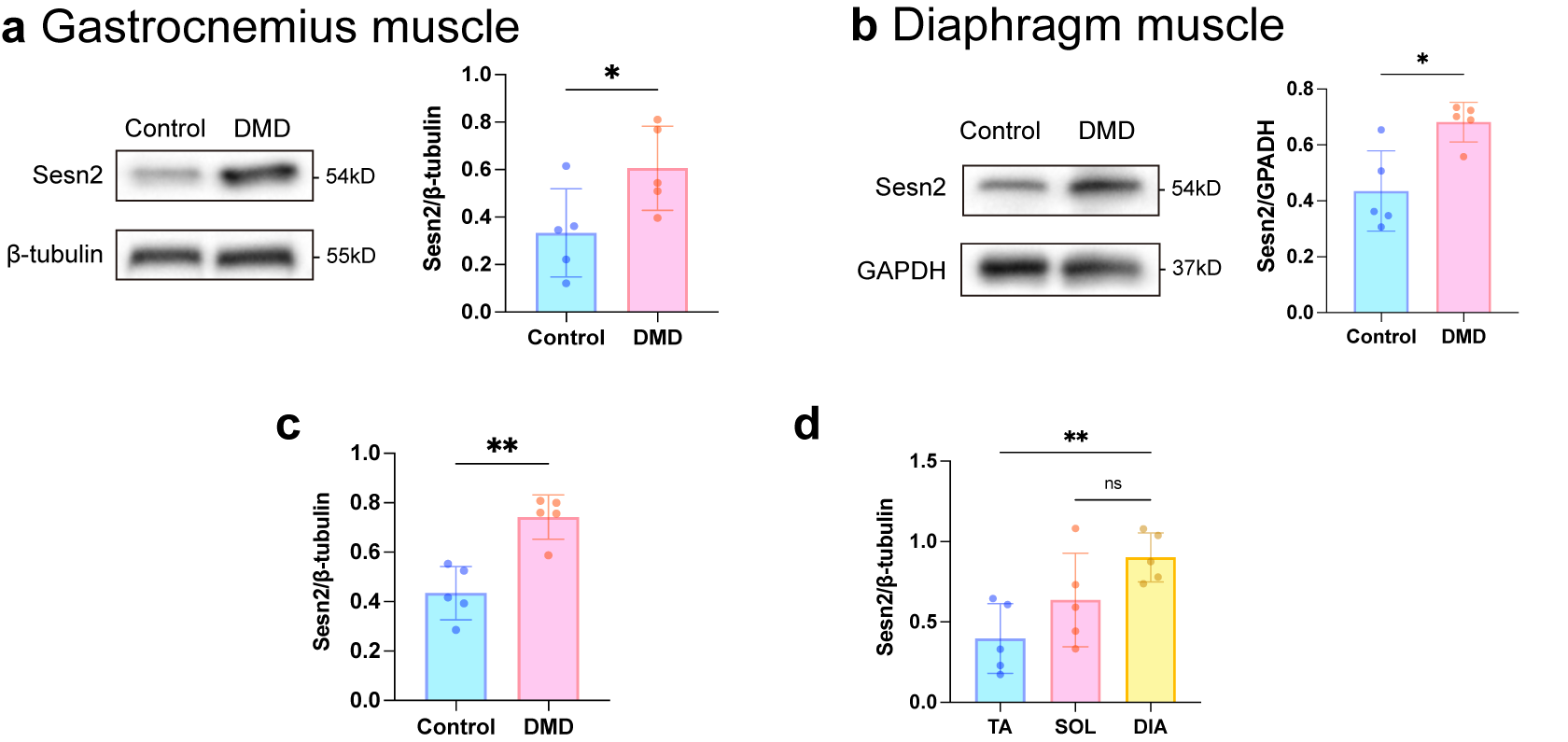


**Fig. S2** Protein expression level of Sesn2 in the gastrocnemius muscle (GAS) and the diaphragm muscle (DIA) of mdx mice. a. Protein expression analysis of Sesn2 in the GAS. Sesn2 was significantly upregulated in the GAS of DMD group. n=5. Student’s test. b. Analysis of Sesn2 protein expression in the DIA. Sesn2 was significantly upregulated in the DIA of DMD group. n=5. Mann-Whitney U test. c. Statistical analysis of protein expression levels in the TA. Statistical analysis results showed that Sesn2 was significantly upregulated in the TA (tibialis anterior muscle, TA) of DMD group. n=5. Student’s test. d. Statistical analysis results indicated that the expression of Sesn2 was highest in the DIA of mdx mice. One-way ANOVA test. n=5. The data are displayed as the mean $\pm$ standard deviation. **p* < 0.05, ***p* < 0.01.


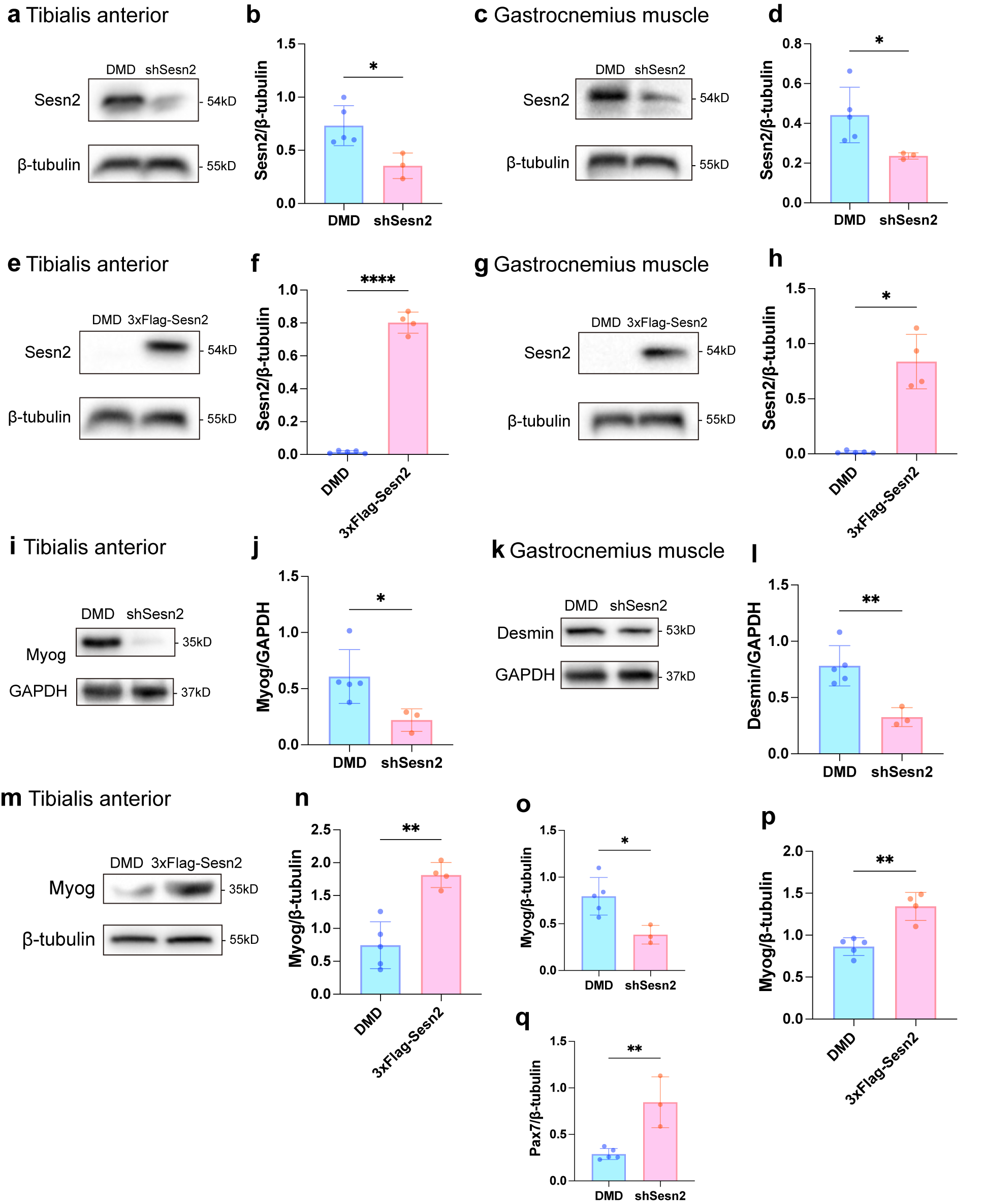


**Fig. S3** Validation of the modulatory effects of Sesn2 overexpression and knockdown. a-d. WB analysis of Sesn2 expression in the TA and GAS in the shSesn2 group. The results showed a significant reduction in the Sesn2 levels, indicating successful knockdown. Student's t test. e-h. WB analysis of Sesn2 expression in the TA and GAS in the 3xFlag-Sesn2 group. The results demonstrated a notable increase in Sesn2 expression, confirming successful overexpression. Statistical analysis of the data in Fig S2f was conducted using student's t test, while the Mann-Whitney U test was used to analyze the data shown in Fig. S2h. i, j. Protein level of Myog in the TA. Myog expression was significantly reduced in the shSesn2 (Sesn2 knockdown) group. Student's t test. k, l. Protein level of Desmin in the GAS. Desmin expression was significantly reduced in the shSesn2 (Sesn2 knockdown) group. Student's t test. m, n. Protein level of Myog in the TA. In the Sesn2 overexpression group (3xFlag-Sesn2 group), Myog was significantly upregulated. Student's t test. o, q. Statistical analysis of the protein expression levels of Myog and Pax7 in the GAS. The results showed that Myog was significantly reduced in the GAS of shSesn2 group, while Pax7 was significantly increased. Student's t test. p. Statistical analysis of the protein expression levels of Myog in the GAS. The results showed that Myog expression was significantly upregulated in the GAS of the 3xFlag-Sesn2 group. Student's t test. The data are displayed as mean standard deviation values. n=3-5. **p* < 0.05, ***p* < 0.01, *****p* < 0.0001.


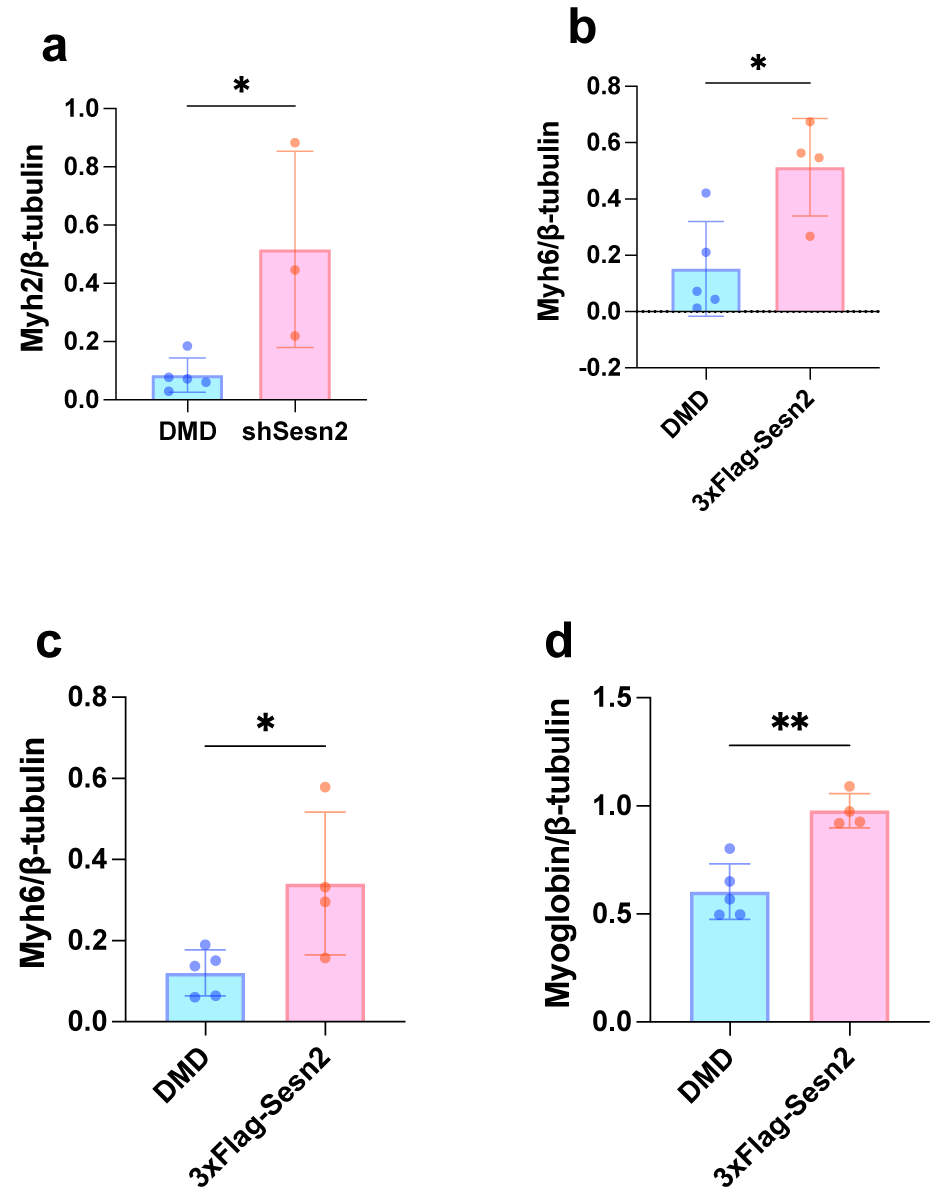


**Fig. S4** Statistical analysis of protein expression levels in Myh2, Myh6 and myoglobin after Sesn2 modulation. a. The protein expression of Myh2 was significantly increased in the TA of shSesn2 group. Student's t test. b, c. In the 3xFlag-Sesn2 group, the protein expression of Myh6 was significantly increased in the GAS (Fig. S4b) and TA (Fig. S4c). Student's t test. d. In the 3xFlag-Sesn2 group, the protein expression of myoglobin was significantly increased in the TA. Student's t test. n=3-5. The data are displayed as mean standard deviation values. **p* < 0.05, ***p* < 0.01.

.

**Table S1**: Primer details

| Gene Symbol | Primer Name | Primer Sequence | Source |
| --- | --- | --- | --- |
| Sesn2 | Forward: | GAGTGCCATTCCGAGATCAAG |  |
|  | Reverse: | TAGTCCGGGTGTAGACCCATC |  |
| Myog | Forward: | GAGACATCCCCCTATTTCTACCA | PrimerBank |
|  | Reverse: | GCTCAGTCCGCTCATAGCC |  |
| Myod | Forward: | ATGATGACCCGTGTTTCGACT | PrimerBank |
|  | Reverse: | CACCGCAGTAGGGAAGTGT |  |
| Myf5 | Forward: | CACCACCAACCCTAACCAGAG | PrimerBank |
|  | Reverse: | AGGCTGTAATAGTTCTCCACCTG |  |
| GPADH | Forward: | TGTGTCCGTCGTGGATCTGA |  |
|  | Reverse: | TTGCTGTTGAAGTCGCAGGAG |  |
| miR-182-5p | Forward: | GGTTTGGCAATGGTAGAACTC |  |
|  | Reverse: | AGTGCAGGGTCCGAGGTATT |  |
|  | Reverse transcription: | GTCGTATCCAGTGCAGGGTCCGAGGTATTCGCACTGGATACGACCGGTGT |  |
| U6 | Forward: | CTCGCTTCGGCAGCACA |  |
|  | Reverse/ Reverse transcription: | GTGCAGGGTCCGAGGT |  |
